# Chitotriosidase: Providing confirmation of cutaneous sarcoidosis when angiotensin converting enzyme fails

**DOI:** 10.1016/j.jdcr.2024.03.012

**Published:** 2024-04-02

**Authors:** Carlie Reeves, Colleen D. Powers, Robert T. Brodell

**Affiliations:** a School of Medicine, University of Mississippi Medical Center, Jackson, Mississippi; b University of Mississippi Medical Center, Jackson, Mississippi; c Department of Pathology, University of Mississippi Medical Center, Jackson, Mississippi; d Department of Dermatology, University of Mississippi Medical Center, Jackson, Mississippi

**Keywords:** lupus pernio, sarcoidosis

## Introduction

Sarcoidosis is a state of granuloma formation that can occur in any organ system. Diagnosis relies on pathologic evidence of granulomas as well as a compatible clinical and radiographic presentation.^1^ The initial symptom is greatly varied, and many patients are asymptomatic, making diagnosis difficult. Chitotriosidase is a serologic marker that can assist in the diagnosis of patients with isolated cutaneous sarcoidosis in the absence of classic radiographic and serologic findings, such as hypercalcemia, elevated angiotensin converting enzyme (ACE) level, or hilar adenopathy. Chitotriosidase (chitinase 1) is a hydrolytic enzyme produced by macrophages that breaks down glycosidic bonds in chitin and plays a role in defense against fungi, insects, and nematodes.^2^ Our case report demonstrates the utility of chitotriosidase in diagnosing a patient with lupus pernio, a type of cutaneous sarcoidosis, when other work-up was inconclusive.

## Case report

**Fig 1:**
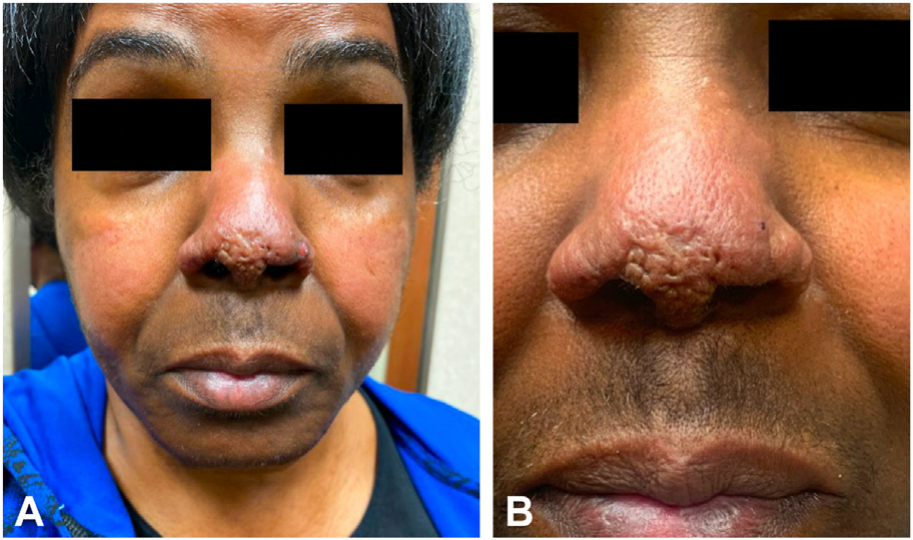
**A,** Indurated papules coalescing in a plaque on the nasal tip and alar rim bilaterally, demonstrating a cobblestone appearance. Erosion on left alar rim consistent with biopsy site. Erythematous to hypopigmented patches on medial aspect of the cheeks, sparing the nasolabial fold. **B,** Atrophic papules on the nasal tip and firm papules along the left nasal ala.

**Fig 2:**
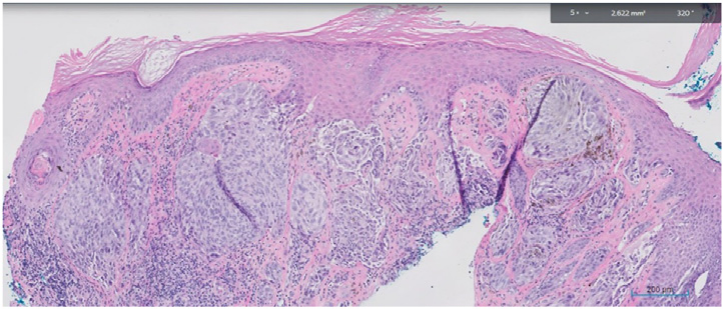
Biopsy of the left nasal ala revealed islands of naked epithelioid granulomas with multinucleated giant cells and lymphocytes throughout the dermis.

A 63-year-old African American woman presented with an 8- to 10-year history of slowly enlarging 2 mm slightly hyperpigmented papules on the tip of the nose and alar rim (Fig 1, *A* and *B*). A shave biopsy of the left nasal ala revealed islands of naked epithelioid granulomas (sarcoidal tubercles) with multinucleated giant cells and a slight admixture of lymphocytes throughout the dermis (Fig 2). Although ACE level was normal at 52 U/L (normal value: 16-85 U/L), chitotriosidase was elevated at 295 nmol/h/mL (normal value: 4.0-120.0 nmol/h/mL). Antinuclear antibody and antineutrophilic cytoplasmic antibodies were normal.

Patient’s past medical history included a secondary spontaneous pneumothorax, with a computed tomography scan demonstrating very mild interstitial lung disease. A bronchoscopy was performed, which demonstrated inconclusive findings. Patient also had a distant 25 pack-year history of smoking.

The diagnosis of lupus pernio was based on her skin findings, sarcoidal granulomas, and elevated chitotriosidase level. Hydroxychloroquine 200 mg twice daily was initiated, and an ophthalmologic examination was scheduled as a baseline for monitoring of hydroxychloroquine retinopathy. Pulmonary function testing revealed moderate restriction and moderate decreased diffusing capacity of the lungs with carbon monoxide. Radiologic imaging demonstrated persistent lymphadenopathy and ground-glass opacities, findings suggestive of active inflammation. Therefore, prednisone was initiated at a dose of 20 mg for 6 weeks. The papules and plaques demonstrated 50% flattening of the cobblestone appearance over 6 months. Repeat chitotriosidase level 7 months after treatment initiation showed improvement to 131 nmol/h/mL.

## Discussion

The percentage of sarcoidosis cases that present with cutaneous involvement varies widely across the literature, likely due to the differences in patient populations. Cutaneous involvement is seen in 20% to 35% of patients with sarcoidosis.^3^ Although erythema nodosum is the most common cutaneous manifestation of sarcoidosis, lupus pernio is the only pathognomonic cutaneous finding.^4^ Lupus pernio is most commonly seen in women aged 45 to 65 years of African descent. Other less common cutaneous manifestations of sarcoidosis include papular sarcoidosis, nodular sarcoidosis, and plaque sarcoidosis.^5^ Because of the wide array of skin presentations and variability in the initial presenting symptoms, the diagnosis is often delayed.^5^ In 20% of patients, skin lesions are the presenting signs of systemic sarcoidosis, but in 30% of patients, the skin lesions appear up to 10 years after the systemic disease has been documented.^3^ Skin and systemic symptoms appear simultaneously in 50% of patients.^3^ The diagnosis of sarcoidosis is often made after lymphadenopathy is noted incidentally on chest x-ray.^6^ Serologic studies can assist in diagnosing sarcoidosis when imaging is inconclusive, as demonstrated in our patient.

Although lupus pernio itself is not life-threatening, initiating treatment promptly is important for curtailing disfigurement. Hydroxychloroquine is the preferred initial therapy for cutaneous sarcoidosis, whereas prednisone is preferred for treating the lungs. In this patient, the diagnosis led to monitoring of pulmonary status through repeat pulmonary function tests and high-resolution computed tomography scans.

The ACE level has been the most frequently used laboratory test for patients with sarcoidosis.^7^ ACE is elevated in 60% of patients with sarcoidosis and is an indication of the total body granuloma burden.^7^ Chitotriosidase can assess disease activity in sarcoidosis with an approximate sensitivity of 88.6% and specificity of 92.8%.^2^ This is in contrast to a sensitivity of 66% and specificity of 54% seen in ACE.^7^ The chitotriosidase test is performed using a blood sample, from which the plasma is separated in a laboratory. The level of chitotriosidase enzyme in the plasma is then measured. It is not routinely available at most hospital laboratories and thus is a send-out test.

Seven months after initiation of treatment in our patient, a repeat chitotriosidase was mildly elevated at 131 nmol/h/mL, which had decreased significantly from 295 nmol/h/mL. A study found that chitotriosidase is seen at its highest levels in patients treated with systemic steroids who have persistent disease.^2^ Concentrations of chitotriosidase decreased dramatically after steroid dose was increased or new immunosuppressant therapy was administered.^2^ As a result, the decrease in chitotriosidase seen in our patient may explain the good response of systemic inflammation to therapy, given that chitotriosidase remained low months after the last dose of prednisone was taken.

This case is novel because it demonstrates the utility of chitotriosidase in diagnosing cutaneous sarcoidosis. The literature on chitotriosidase has only been in reference to systemic sarcoidosis, not its cutaneous manifestations. In addition, there is very little mention of chitotriosidase in the dermatologic literature. One article found chitotriosidase to be a marker of systemic inflammation in psoriasis, but associations between chitotriosidase and cutaneous sarcoidosis have not been reported.^8^ Because skin lesions are the manifesting symptoms in 20% of patients with sarcoidosis, chitotriosidase is a marker that dermatologists should use in the diagnostic work-up for cutaneous sarcoidosis.

## Conflicts of interest

None disclosed.
